# Suppression of IκBα increases the expression of matrix metalloproteinase-2 in human ciliary muscle cells

**Published:** 2009-09-26

**Authors:** Yu-Qing Lan, Chi Zhang, Jian-Hui Xiao, Ye-Hong Zhuo, Hui Guo, Wei Peng, Jian Ge

**Affiliations:** 1 Department of Ophthalmology, The Second Affiliated Hospital, Sun Yat-sen University, Guangzhou, Guangdong, China; 2 Zhongshan Ophthalmic Center, Sun Yat–sen University, Guangzhou, Guangdong, China

## Abstract

**Purpose:**

An increase of matrix metalloproteinase-2 (MMP-2) has been found to improve outflow through the uveoscleral pathway. This experiment was designed to test whether reduction of inhibitor of nuclear factor kappa B alpha (IκBα) levels could enhance MMP-2 expression in human ciliary muscle (HCM) cells in vitro.

**Methods:**

The small interfering RNA (siRNA) targeting inhibitor of nuclear factor kappa B (*IκBα*) was transfected into HCM cells. The mRNA and protein levels of IκBα, nuclear factor-kappa B (NF-κB)p65, MMP-2, tissue inhibitor of metalloproteinase-2 (TIMP-2), and membrane-type 1 matrix metalloproteinase (MT1-MMP) in HCM cells were examined 24 h, 48 h, and 72 h after *IκBα* siRNA transfection by real-time reverse transcription polymerase chain reaction (RT–PCR) and western blot. The activation of NF-κBp65 was determined through the translocation of NF-κBp65 by fluorescence microscope. Gelatin zymography was used to detect the secretion and activity of MMP-2.

**Results:**

Real-time RT–PCR and western blot showed that transfection of *IκBα* siRNA led to gradual downregulation of IκBα and TIMP-2 both at the mRNA and protein level after 24 h, 48 h and 72 h. The *IκBα* and *TIMP-2* mRNA levels decreased 92.7%±1.6% and 70.3%±13.1%, respectively, and the protein levels were reduced 87.3%±2.0% and 62.9%±0.8% (p<0.01), respectively, when compared to the control 72 h after siRNA transfection. Conversely, the MMP-2 and MT1-MMP mRNA and protein levels increased in the time-dependent manner after *IκBα* siRNA transfection. The *MMP-2* and *MT1-MMP* mRNA levels increased 178%±4.6% and 165%±8.2%, respectively, while protein levels were raised to 162%±3.7% and 157.6%±5.7% (p<0.01), respectively, when compared to the control  72 h after *IκBα* siRNA transfection. Although no obvious changes were seen in either mRNA or protein levels of total NF-κBp65 *(*p>0.05), the protein level of NF-κBp65 increased dramatically in the nucleus as revealed by western blot and fluorescence staining 24 h, 48 h, and 72 h after *IκBα* siRNA transfection. Moreover, gelatin zymography indicated that the secretion and activity of MMP-2 in treated cells were higher than those in the control cells. The maximum increases of pro-MMP-2 and active-MMP-2 were 172%±15% and 151%±14% (p<0.01), respectively, when compared to the control at the experiment’s conclusion 72 h after siRNA transfection.

**Conclusions:**

Expression and activity of MMP-2 was enhanced by the *IκBα* siRNA in HCM cells through the activation of the NF-κB signaling pathway. Our results suggested that *IκBα* may therefore be a potential target for controlling the uveoscleral outflow pathway in glaucoma.

## Introduction

In glaucomatous primates [1] and humans [2], prostaglandin (PG)F2α has been shown to lower the intraocular pressure (IOP), which results from increased uveoscleral outflow. Although the precise mechanism by which prostaglandins improve uveoscleral outflow is not fully understood, two possible mechanisms that have been studied are relaxation of the ciliary muscle and remodeling the extracellular matrix of the ciliary muscle [3,4]. Researchers have attributed the increase in uveoscleral outflow to a group of enzymes named matrix metalloproteinases (MMPs) [5,6].

MMPs are zinc-dependent endopeptidases that play a key role in regulating the modulations of the extracellular matrix (ECM). These molecules are secreted much like proenzymes and become activated by proteolytic cleavage truncation [7]. MMP-2, a member of the family of MMPs, has been shown to have proteinase activity in the degradation of multiple ECM proteins such as type I collagen, laminin, fibronectin, and several proteoglycans [8,9]. MMP-2 is secreted as an inactive proenzyme, pro-MMP-2. The activation of pro-MMP-2 requires its cell surface localization and cleavage by cell membrane-type 1 matrix metalloproteinase (MT1-MMP), which controls a variety of physiologic and pathological processes through the proteolytic degradation of extracellular or transmembrane proteins [10]. Tissue inhibitor of metalloproteinase-2 (TIMP-2), the specific inhibitor of MMP-2, is also involved in this process by forming a receptor for pro-MMP-2 [11]. It has been confirmed that the extracellular spaces separating ciliary muscle fibers of the uveoscleral outflow pathway contain abundant ECM [12,13]. Thus, increased degradation of ciliary muscle ECM by MMP-2 could remodel the ECM among the ciliary muscle bundles and decrease the hydraulic resistance to uveoscleral flow [14].

Nuclear factor-kappa B (NF-κB) is one of the major transcription factors that plays a critical role in the gene regulation of multiple cellular processes such as inflammation, immune responses, cell proliferation, and apoptosis [15,16]. Five members were identified among the NF-κB family of transcription factors: v-rel reticuloendotheliosis viral oncogene homolog (Rel)A (p65), RelB, NF-κB1 (p50/p105), NF-κB2(p52/p100), and c-Rel [17]. In most cells, NF-κB forms various homodimers as well as heterodimers, mainly between the p65 and p50 proteins. The NF-κB dimers can form complexes in the cytoplasm with the inhibitor of nuclear factor kappa B alpha (IκBα), which masks the NF-κB nuclear localization sequence and results in latent inactivity. However, upon activation by extracellular stimuli or other transcriptional factors, IκBα is phosphorylated and degraded while NF-κB rapidly translocates to the nucleus where it activates the transcription expression of several important genes [18,19].

Several studies have documented that the suppression of IκBα could induce NF-κB nuclear translocation and consequently promote the increase of MMP-2 in several types of human cells including Ewing sarcoma EW7 cells [20], human skin cells [21], murine melanoma cells [22], and HT1080 (fibrosarcoma) cells [23]. However, the molecular mechanism of inducing the secretion and activation of MMP-2 in human ciliary muscle (HCM) cells still remains unclear. This question has led us to investigate whether the IκB/NF-κB signaling pathway influences MMP-2 expression in HCM cells in vitro.

## Methods

### Primary HCM cells

Normal primary HCM cells were established and cultured as described previously by the Declaration of Helsinki [24]. HCM cells were prepared from eight donors with no history of eye diseases (five males, three females) around 18–24 h after death and were obtained from Zhongshan Ophthalmic Center (Sun Yat-sen University, Guangzhou, China) using a procedure described earlier [25]. The cells used in the present experiments were passaged four to six times to minimize the dedifferentiation that might occur in late passage. The HCM cells were further identified with monoclonal rabbit anti-smooth muscle α-actin and monoclonal rabbit anti-Desmin (Sigma, St. Louis, MO) by immunocytochemical staining.

### Fluorescence immunostaining

HCM cells were grown on coverslips in six-well plates. After 24 h, the cells were washed three times with PBS, fixed with 4% formaldehyde, blocked in fresh 1% BSA/PBS for 1 h, and stained with monoclonal anti-smooth muscle α-actin (1:100; Sigma) and rhodamine-conjugated goat anti-rabbit IgG (1:50; Sigma). The nuclei of the cells were counterstained with 4',6'-diamidino-2-phenylindole dihydrochloride (DAPI, 1:1000; Sigma). Control cells were performed using PBS instead of the primary antibody. Stained cells were viewed with the Axioplan2 fluorescence microscope (Carl Zeiss, Oberkochen, Germany). HCM cells were stained with monoclonal rabbit anti-NF-κBp65 (1:50; BD, Franklin Lakes, NJ) and FITC-conjugated goat anti-rabbit IgG (1:500; BD) by the procedure described above 24 h, 48 h, and 72 h after *IκBα* small interfering RNA (siRNA) transfection. HCM cells were stained with monoclonal rabbit anti-Desmin (1:100) and goat anti-rabbit IgG (1:50) according to the instructions included with the SP-9001 Histostain-Plus Kit (Beijing Zhongshan Gold Bridge, Beijing, China).

### Preparation and transfection of siRNA

The siRNA sequences used for silencing *IκBα* (GenBank NM_020529) were designed, and Cy3-labled *IκBα* siRNA was synthesized by Guangzhou Ribobio (Guangzhou, China).The sequences of siRNAs were as follows: sense, 5′-CUC CGA GAC UUU CGA GGA A dTdT-3′; antisense, 5′-UUC CUC GAA AGU CUC GG AG dTdT-3′. For nonsense control siRNA, we used irrelevant siRNA with random nucleotides and no known specificity (Qiagen, Hilden, Germany). Its sequences were as follows: sense, 5′-UUC UCC GAA CGU GUC ACG UdTdT-3′; antisense, 5′-ACG UGA CAC GUU CGG AG A AdTdT-3′. HCM cells (3×10^4^) were plated in a six-well plate, incubated for 24 h, and transfected with a *IκBα*-specific siRNA duplex (50 nM final concentration) or the nonsense control siRNA (50 nM) using the Lipofectamine 2000 reagent (Invitrogen, Carlsbad, CA) according to the manufacturer’s instructions.

### Transfection efficiency of siRNA

To monitor transfection efficiency, the HCM cells were transfected with Cy3-labled *IκBα* siRNA in a six-well plate. The cells were washed twice with PBS 24 h, 48 h, and 72 h after *IκBα* siRNA transfection and then viewed by the Axioplan2 fluorescence microscope (Carl Zeiss). Transfection efficiency was determined by counting the fraction of fluorescing cells in 10 fields of view.

### Real-time reverse transcription polymerase chain reaction analysis

Total RNA was extracted from HCM cells with an RNeasy kit (Qiagen, Hilden, Germany) 24 h, 48 h, and 72 h after *IκBα* siRNA transfection. The extracted RNA was pretreated with RNAase-free DNase, and 0.2 μg of RNA from each sample was used for cDNA synthesis primed with random hexamers. Real-time reverse transcription polymerase chain reaction (RT–PCR) was performed on six genes, *NF-κBp65*, *IκBα*, *MMP-2*, *TIMP-2*, *MT1-MMP*, and *GAPDH* (internal control). The real-time RT–PCR primers are summarized in Table 1. Real-time RT–PCR was performed in a total volume of 20 μl from a mixture containing 10 μl of SYBR1 Premix Ex Taq (Takara, Tokyo, Japan), 0.4 μl of forward primer (10 mM), 0.4 μl of reverse primer (10 mM), 0.4 μl of ROX Reference Dye (50×; Takara, Tokyo, Japan), 6.8 μl of dH_2_O, and 2 μl of template cDNA solution. Reactions were run in an ABI PRISM7000 Sequence Detector system (Applied Biosystems, Foster City, CA) under the following conditions: the PCR was run for 40 cycles of 95 °C for 5 s, 56 °C for 15 s and 72 °C for 31s after pre-incubation at 95°C for 10 s. All reactions were performed in duplicate. The specificity of the amplification reactions was confirmed by melting-curve analysis. Relative mRNA expression levels of the six genes in the siRNA-treated samples and controls were determined using the comparative threshold (C_t_) method and analyzed with Sequence Detector software version 1.7 (Applied Biosystems). The fold changes in the mRNA expression level of *IκBα*, *NF-κBp65*, *MMP-2*, *TIMP-2*, and *MT1-MMP* in siRNA-treated and control samples were compared using the 2^-ΔΔCt^ method [26].

**Table 1:** Nucleotide sequences of human specific primers for real-time RT–PCR.

**Gene**	**Primers**	**Sequence**	**Product size (bp)**
*IκBα*	Forward	5′-AAGTGATCCGCCAGGTGAAG-3′	86
	Reverse	5′-GCAATTTCTGGCTGGTTGGT-3	
*NF-κBp65*	Forward	5′-TCTCCCTGGTCACCAAGGAC-3′	64
	Reverse	5′-TCATAGAAGCCATCCCGGC-3′	
*MMP-2*	Forward	5′-CGAATCCATGATGGAGAGGC-3′	87
	Reverse	5′-TCCGTCCTTACCGTCAAAGG-3	
*GAPDH*	Forward	5′-CACCAACTGCTTAGCACCCC-3′	81
	Reverse	5′-TCTTCTGGGTGGCAGTGATG-3′	
*TIMP-2*	Forward	5′-TTCATTCGTCTCCCGTCTTT-3′	113
	Reverse	5′-ACCAACGTGTGTGGATCAAA-3′	
*MT1-MMP*	Forward	5′-TCGGCCCAAAGCAGCAGCTC-3	180
	Reverse	5′-CTTCATGGTGTCTGCATCAGC-3′	

The oligonucleotide sequences of real-time RT-PCR primers for Human IκBα, NF-κBp65, MMP-2, GAPDH, TIMP-2, and MT1-MMP are shown in the table.

### Western blot analysis

Total subcellular and nuclear proteins in different groups were extracted by the Nuclear Extract Kit (Active Motif, Carlsbad, CA) 24 h, 48 h, and 72 h after *IκBα* siRNA transfection, according to the manufacturer’s instructions. Briefly, cells were scraped, washed in PBS, and centrifuged for 5 min at 700× g at 4 °C. The supernatant was discarded, and cells were resuspended in a hypotonic buffer and incubated for 15 min on ice. Detergent was added and the suspension vortexed for 10 s. Next, the suspension was centrifuged for 30 s at 14,000× g, and the nuclear pellet was resuspended in 50 μl of complete lysis buffer. The suspension was incubated for 30 min on ice on a rocking platform set at 140× g and centrifuged for 10 min at 14,000× g at 4 °C. The supernatant was used as nuclear extract. The protein concentration was measured by the Bio-Rad protein assay (Bio-Rad, Hercules, CA). The total protein extracts (30 μg each) were separated from each sample by 10% sodium dodecyl sulfate polyacrylamide gel electrophoresis (SDS–PAGE) and transferred onto poly vinylidene fluoride (PVDF) membranes. The membranes were blocked with 5% blocking reagent at room temperature for 1.5 h and probed with polyclonal rabbit anti-IκBα (1:1,000; Cell Signaling Technology, Danvers, MA), polyclonal rabbit anti-MT1-MMP (1:1,000; Santa Cruz Biotechnology, Santa Cruz, CA), and polyclonal rabbit anti-GAPDH (1:5,000; BD) overnight at 4 °C. The membranes were washed three times with the blocking buffer and incubated with goat anti-rabbit IgG conjugated with horseradish peroxidase (HRP; BD). The bands were visualized with an enhanced chemiluminescence kit (ECL kit; Cell Signaling Technology). The protein level of NF-κBp65 in both the total (30 μg) and nuclear (30 μg) extracts was detected by western blot analysis using polyclonal rabbit anti-NF-κBp65 (1:1,000; Cell Signaling Technology). MMP-2 and TIMP-2 expression was examined as follows: the medium was replaced with serum-free growth medium after transfection for 6 h. The conditioned medium was then collected and concentrated by centrifugal ultrafiltration (Amicon, Houston, TX) 24 h, 48 h, and 72 h after *IκBα* siRNA transfection and stored at −80 °C. An equal amount (30 μg) of total proteins was concentrated from the medium to detect the expression of MMP-2 and TIMP-2 with polyclonal rabbit anti-MMP-2 (1:1,000; Cell Signaling Technology) and polyclonal rabbit anti-TIMP-2 (1:1,000; Santa Cruz Biotechnology) according to the procedures described above. The band intensities were quantified by Gel-pro Analyzer 4.0 (Media Cybernetics, Silver Spring, MD).

### Gelatin zymography analysis

The conditioned medium was collected by centrifugation and concentrated 24 h, 48 h, and 72 h after *IκBα* siRNA transfection. Protein concentrations were measured using the Bio-Rad protein assay. The samples containing an equal amount of total proteins (30 μg) were mixed with sample buffer in the absence of a reducing agent, incubated at room temperature for 30 min, and loaded onto 10% SDS–PAGE containing 0.1% gelatin (Amersco, St. Louis, MO). After electrophoresis, gels were washed twice in 50 mM Tris-HCl buffer (pH 7.5), which contained 2.5% Triton X-100, for 30 min followed by incubation in an activation buffer (50 mM Tris-HCl [pH 7.5], 200 nM NaCl, 1 μM ZnCl_2_, and 10 mM CaCl_2_) for 24 h at 37 °C until enzymatic degradation of the substrate took place. Gels were stained with 0.1% Coomassie blue R-250 (Sigma, St. Louis, MO) and then destained. Gelatinolytic bands were observed as clear zones against the blue background, and the intensity of the bands was quantified by Gel-pro Analyzer 4.0 (Media Cybernetics) to provide a semi-quantitative assay of the enzymatic activity.

### Statistical analysis

All results were presented as mean±standard deviation (SD). Statistical significance was assessed with one-way analysis of variance (ANOVA). A p<0.05 was considered statistically significant.

## Results

### Establishment of primary HCM cells

**Figure 1:**
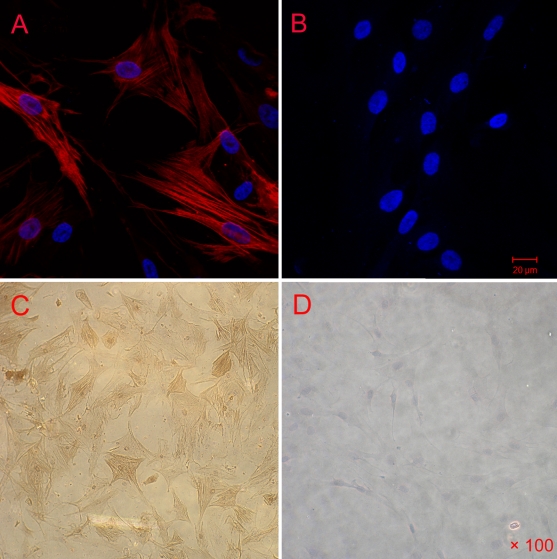
Identification of HCM cells. The third passaged HCM cells were washed with PBS, fixed with formaldehyde, blocked in BSA/PBS and stained with monoclonal anti-smooth muscle α-actin and rhodamine-conjugated goat anti-rabbit IgG;HCM cells were also stained with monoclonal rabbit anti-Desmin and goat anti-rabbit IgG. HCM cells labeled with antibody to smooth muscle actin (**A**; red) and the negative control (**B**). Nuclei staining with DAPI is shown in blue. HCM cells labeled with antibody to desmin (Buffy; **C**) and the negative control (**D**).

HCM cells were prepared from normal human donors with no history of ocular diseases. It takes about four to eight days for the primary HCM cells to form a monolayer of spindle-shaped cells with characteristic “hill-and-valley patterns” [27,28].The HCM cells were identified by the positive staining of smooth muscle α-actin and Desmin by immunocytochemistry (Figure 1A,C). Negative control cultures showed no positive staining (Figure 1B,D), demonstrating that the cells we isolated were pure HCM cells.

### Transfection efficiency of IκBα siRNA

**Figure 2:**
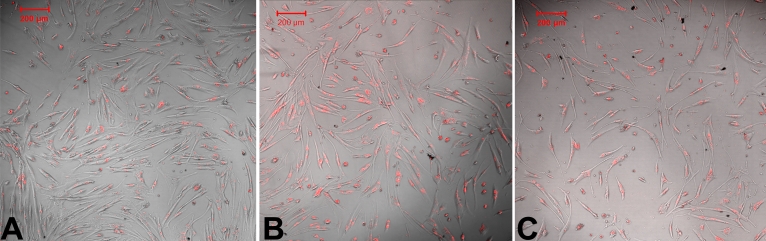
Transfection efficiency of IκBα siRNA. To monitor siRNA transfection efficiency, the HCM cells were transfected with Cy3-labled IκBα siRNA using Lipofectamine 2000 reagent, which were viewed at 24 h, 48 h, and 72 h after transfection. Cy3-labled *IκBα* siRNA (red) was observed in the cytoplasm in HCM cells, and the transfection efficiency was 92% (**A**), 90% (**B**), and 91% (**C**) 24 h, 48 h, and 72 h after *IκBα* siRNA transfection, respectively.

Transfection efficiency of *IκBα* siRNA 24 h, 48 h, and 72h after transfection (determined by fluorescence microscopy) was 92%, 90%, and 91%, respectively (Figure 2A-C).

### Suppression of IκBα mRNA and protein expression by siRNA in HCM cells

**Figure 3:**
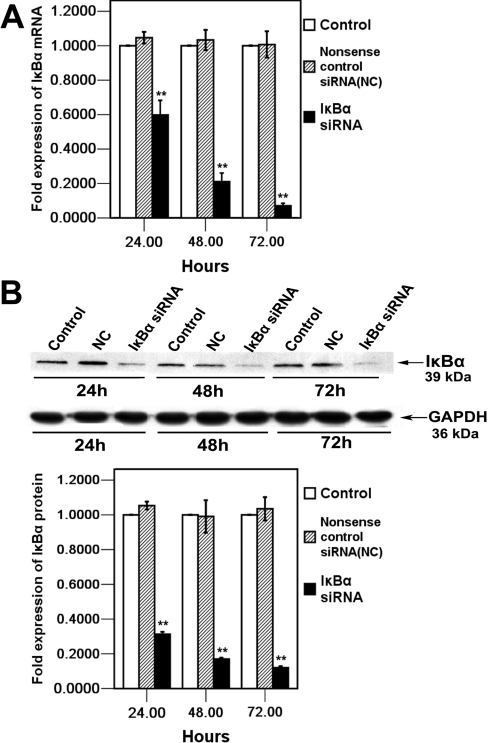
Examination of *IκBα* mRNA and protein levels in HCM cells 24 h, 48 h, and 72 h after *IκBα* siRNA transfection. **A**: *IκBα* mRNA expression was quantified by real-time RT–PCR. Expression levels were normalized with *GAPDH*. Error bars represent standard deviations (SD) calculated from three parallel experiments. **B**: Total cell lysates from HCM cells treated with *IκBα* siRNA, nonsense control siRNA (NC), and control were analyzed by western blot with IκBα antibody and GAPDH antibody. The arrows indicate IκBα (39 kDa) and GAPDH (36 kDa) bands. The bands were analyzed densitometrically, and the values were normalized with GAPDH, which are represented in the bar graph. The mRNA and protein values of IκBα siRNA, control, and nonsense control siRNA (NC) groups were determined by one-way ANOVA. The double asterisk denotes p<0.01.

We hypothesized that the IκBα pathway plays a role in MMP-2 expression in HCM cells. Real-time RT–PCR analysis was performed to examine the *IκBα* mRNA level after specific *IκBα* siRNA transfection at 24 h, 48 h, and 72 h. As shown in Figure 3A, *IκBα* mRNA levels were markedly suppressed at each time point to 41.1%±8.3% at 24 h, 78.7%±4.8% at 48 h, and 92.7%±1.6% (mean±SD, n=4) at 72 h, respectively, compared to those in control after *IκBα* siRNA transfection. Furthermore, the western blot analysis indicated dramatic reduction of IκBα protein in the *IκBα* siRNA-treated cells in a time dependent manner, and the IκBα protein levels were suppressed significantly to 69.8%±1.3% at 24 h, 83.1±1.6% at 48 h, and 87.3%±2.0% (mean±SD, n=3) at 72 h, respectively, compared to those in control after *IκBα* siRNA transfection (Figure 3B). Taken together, these results indicated that IκBα mRNA and protein were effectively repressed by *IκBα* siRNA in cultured HCM cells.

### Effects of *IκBα* siRNA on MMP-2 expression in HCM cells

**Figure 4:**
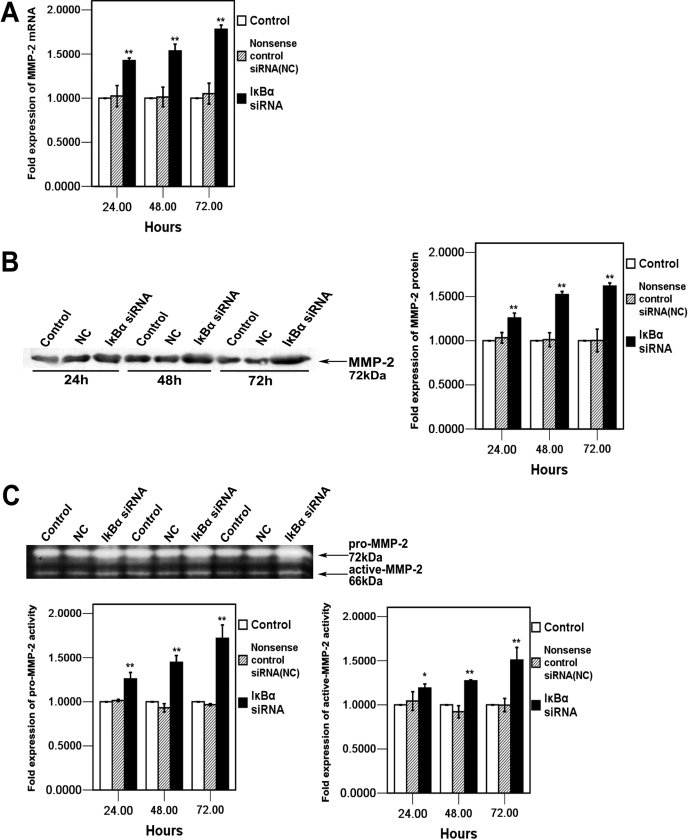
Effect of ablation of *IκBα* on MMP-2 expression and activity 24 h, 48 h, and 72 h after *IκBα* siRNA transfection. **A**:*****MMP-2* mRNA expression in in HCM cells of IκBα siRNA transfected, nonsense control siRNA (NC) transfected, and control was quantified by real-time RT-PCR. Expression levels were normalized with *GAPDH*. **B**: After *IκBα* siRNA transfection, the conditioned media were collected at the indicated time, concentrated, and analyzed by western blot with MMP-2 antibody. The arrow indicates MMP-2 bands that are analyzed by densitometry and the values were represented in the bar graph. **C**: The activity of MMP-2 is analyzed by gelatin zymography analysis. The arrows indicate pro-MMP-2 (72-kDa) and active MMP-2 (66-kDa) specific bands. The bands were analyzed by densitometry and are represented in the bar graph. The mRNA, protein, and activity values of IκBα siRNA transfected, nonsense control siRNA (NC) transfected, and control were determined by one-way ANOVA. An asterisk denotes p<0.05, and a double asterisk indicates p<0.01.

In addition to studying IκBα level, we also analyzed MMP-2 expression in HCM cells. To investigate whether the knockdown of IκBα can upregulate MMP-2, the mRNA level of *MMP-2* was examined by real time RT–PCR analysis. As shown in Figure 4A, the mRNA level of *MMP-2* increased after *IκBα* siRNA transfection in a time-dependent manner and reached to the highest level at 178%±4.6% (mean±SD, n=4) in HCM cells at 72 h. Furthermore, western blot analysis indicated that the protein level of MMP-2 also increased in a time-dependent manner and reached up to 162%±3.7% (mean±SD, n=3) compared to those in control 72 h after *IκBα* siRNA transfection, which was demonstrated by densitometry analysis (Figure 4B). Apart from studying mRNA and protein levels, we also performed a functional assay. Two major gelatinolytic activities, which corresponded to the pro-MMP-2 form (72 kDa) and active-MMP-2 form (66 kDa), were observed on a gelatin zymogram assay. *IκBα* siRNA transfection increased the levels of both pro- and active-MMP-2 in HCM cells in a time-dependent manner when compared with nonsense control siRNA (NC) and control cells after *IκBα* siRNA transfection. Densitometry analysis indicated that the maximum increases achieved for both pro- and active-MMP-2 were 172%±15% and 151%±14% (mean±SD, n=3), respectively (Figure 4C).

### Effects of *IκBα* siRNA on TIMP-2 and MT1-MMP expression in HCM cells

**Figure 5:**
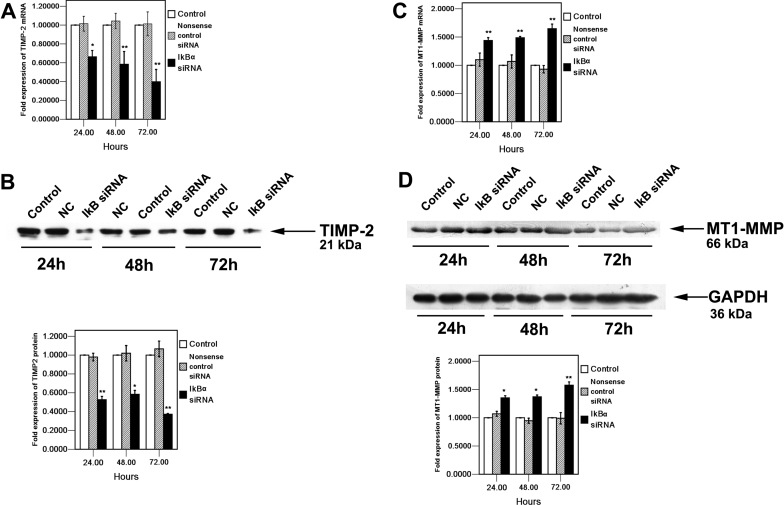
Effect of knockdown IκBα on TIMP-2 and MT1-MMP expression in HCM cells 24 h, 48 h, and 72 h after *IκBα* siRNA transfection. *TIMP-2* (**A**) and *MT1-MMP* (**C**) mRNA expression in HCM cells of IκBα siRNA transfected, nonsense control siRNA (NC) transfected, and control was quantified by real-time RT-PCR. Expression levels were normalized with *GAPDH*. **B**: After *IκBα* siRNA transfection, the conditioned media were collected at the indicated time, concentrated, and analyzed by western blot with TIMP-2 antibody. The arrow indicates TIMP-2 (21 kDa). The bands were analyzed by densitometry and represented in the bar graph. **D**: Total cell lysates from HCM cells transfected with IκBα siRNA , nonsense control siRNA (NC) as well as  from the control, respectively, were analyzed by western blot with MT1-MMP antibody. The arrows show MT1-MMP (66 kDa) and internal control, GAPDH (36 kDa). The bands were analyzed by densitometry and represented in the bar graph. The mRNA, protein, and activity values of IκBα siRNA transfected, nonsense control siRNA (NC) transfected, and control were determined by one-way ANOVA. An asterisk denotes p<0.05, and a double asterisk indicates p<0.01.

The *TIMP-2* mRNA level was suppressed at each time point to 33.7%±6.7% at 24h, 41.7%±13.6 at 48 h and %70.3%±13.1% (mean±SD, n=4) at 72 h, respectively, compared to those in control after *IκBα* siRNA transfection (Figure 5A). Furthermore, western blot analysis indicated the TIMP-2 protein level in the *IκBα* siRNA-treated cells was reduced significantly to 62.9%±0.8% compared to those in control at 72 h after *IκBα* siRNA transfection (Figure 5B). As shown in Figure 5C, the mRNA level of *MT1-MMP* increased in a time-dependent manner and reached to the highest level 72 h after *IκBα* siRNA transfection at 165%±8.18% (mean ± SD, n=4) in HCM cells. Furthermore, western blot analysis indicated that the protein level of MT1-MMP also increased in a time dependent manner to 135.2%±3.7% at 24h, 137.1%±3.2% at 48h and 157.6%±5.7% (mean±SD, n=3) at 72 h, respectively, compared to those in control after IκBα siRNA transfection, which was demonstrated by the densitometric analysis (Figure 5D).

### Effect of *IκBα* siRNA on NF-κBp65 expression and nuclear translocation in HCM cells

**Figure 6:**
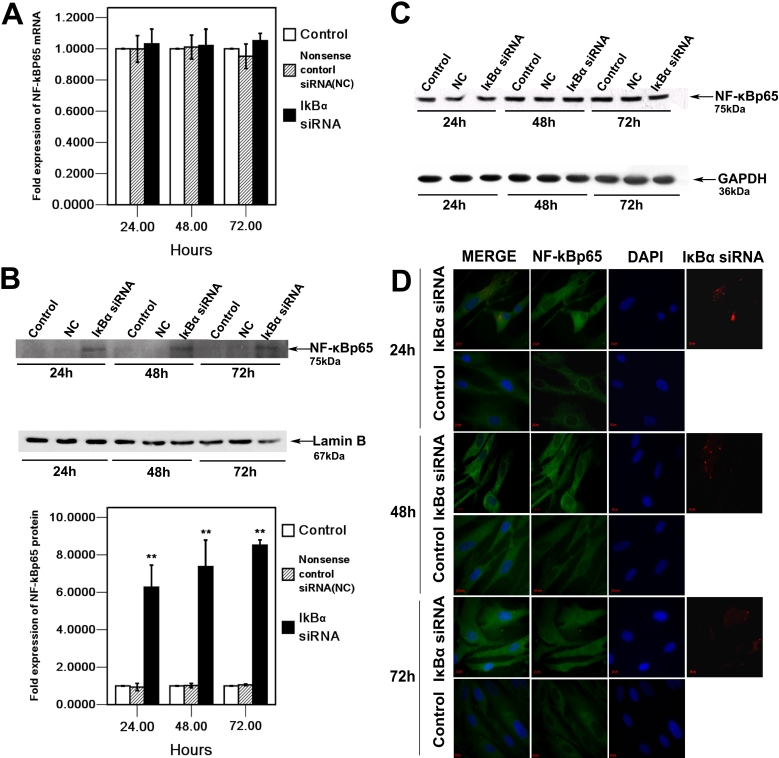
Effect of knockdown IκBα on the expression and cellular localization of NF-κBp65 in HCM cells 24 h, 48 h, and 72 h after *IκBα* siRNA transfection. **A**: *NF-κBp65* mRNA expression in HCM cells of IκBα siRNA transfected, nonsense control siRNA (NC) transfected, and control was quantified by real-time RT-PCR. Expression levels were normalized with *GAPDH*. **B**: Nuclear proteins extracted from IκBα siRNA, nonsense control siRNA (NC), as well as control cells, respectively, were analyzed by western blot with NF-κBp65 antibody. The arrow indicates the NF-κBp65 (75 kDa) band and nucleus internal control, Lamin B (67 kDa). The bands were analyzed densitometrically, and the values were normalized with Lamin B, represented in bar graph. **C**: Total cell lysates from HCM cells transfected with IκBα siRNA, and nonsense control siRNA (NC) as well as  from the control, respectively, were analyzed by western blot with NF-κBp65 antibody. The arrows show the NF-κBp65 (75 kDa) band and the internal control, GAPDH (36 kDa). The mRNA and protein values compared to the control and nonsense control siRNA (NC) were determined by one-way ANOVA. The double asterisk denotes p<0.01. **D**: The HCM cells were immunostained with NF-κBp65 antibody and analyzed by fluorescence microscopy. A weak nuclear signal of NF-κBp65 (green) was observed in control cells at 24 h, 48 h, and 72 h. After *IκBα* siRNA transfection, NF-κBp65 translocated from the cytoplasm into the nucleus, and a strong signal of NF-κBp65 was detected in the nucleus at 24 h, 48 h, and 72 h. Cy3 labled IκBα siRNA (red) was observed in the cytoplasm. Cell nuclei were counterstained with DAPI (blue).

To further investigate the molecular mechanism that explains how the knockdown of IκBα affects the secretion and activation of MMP-2, the NF-κB, which is in the downstream pathway of IκBα, was examined in HCM cells. Real-time RT–PCR and western blot analysis indicated that there were no significant differences between NF-κBp65 mRNA and protein levels at each time point (Figure 6A,C). Nevertheless, the expression of NF-κBp65 in the nucleus was markedly increased in HCM cells after *IκBα* siRNA transfection when compared to those in the control cells (Figure 6B). Moreover, immunofluorescent staining demonstrated that silencing of IκBα could induce translocation of NF-κBp65 into the nucleus. As shown in Figure 6D, NF-κBp65 staining mainly localizes in the nucleus 24 h, 48 h, and 72 h after *IκBα* siRNA transfection in HCM cells whereas little nuclear translocation of NF-κBp65 was observed in control HCM cells.

## Discussion

In the current study, our results demonstrated that the IκB/NF-κB signaling pathway was involved in the expression and activation of MMP-2 in human ciliary muscle cells. Downregulation of *IκBα* by siRNA resulted in significant translocation of NF-κBp65 from the cytoplasm to the nucleus after 24 h to 72 h. The secretion of MMP-2 increased as well as the activation from pro-MMP-2 to active-MMP-2. In addition, the expression of MT1-MMP increased while the expression of TIMP-2 reduced both in mRNA and protein levels. It is implied from these results that NF-κB is an important transcriptional factor involved in the control of uveoscleral outflow through the promotion of MMP-2 expression.

It has been reported that NF-κB is involved in various activities and plays a key role in multiple biological processes including chemical stress, physical stress, physiologic stress, receptor ligands, proinflammatory cytokines, and apoptosis. At the same time, there are also multiple and complex signal transduction pathways leading to NF-κB activation. However, many of these activations are primarily performed by the so-called classical pathway, which involves the degradation of IκBα, leading to the release of the NF-κB complex, which in turn allows it to relocate to the nucleus [29]. Meanwhile, mounting evidence shows that the IκB/NF-κB classical pathway is involved in the expression of MMP-2. Han et al. [21] reported that TNF-α induced the breakdown of IκB in fibroblasts within the collagen lattice, which is a critical step leading to the activation of pro-MMP-2 by NF-κB. Philip and Kundu [22] showed that osteopontin could induce NF-κB activity through phosphorylation and degradation of IκBα by activating I-Kappa B Kinase (IKK), which ultimately triggers the activation of pro-MMP-2 in B16F10 cells. Park and his colleagues [23] indicated that propylene glycol monomethyl acetic ether (PMA) treatment resulted in IκBα phosphorylation in fibrosarcoma cells, which enhances MMP-2 production through the activation of NF-κB transcription factors.

To further investigate the potential role of NF-κB in uveoscleral outflow, the expression of *IκBα* was downregulated by specific siRNA. We found not only the translocation of NF-κBp65 from the cytoplasm into the nucleus but also the enhanced secretion and activity of MMP-2 after IκBα knockdown. Most MMPs are secreted in their inactive pro-form from cells and in the active-form outside the cells. Many studies have indicated that the secretion [30] and activation of MMP-2 [21,22] are associated with NF-κB . On the other hand, NF-κB signaling has also been demonstrated to participate in the regulation of MMP-2 at a transcriptional level as shown in our study, indicating that *MMP-2* mRNA increases after NF-κB has been activated. It has been reported that PGF2α induces c-Fos in human ciliary smooth muscle cells [31] and that c-Fos is capable of physically interacting with NF-κBp65 through the Rel homology domain, enhancing its DNA binding and biological function [32]. Moreover, although there is no NF-κB-binding site on the *MMP-2* promoter, it has been suggested that MMP-2 activation occurs in endothelial cells through an NF-κB-dependent pathway [30]. Apparently, the molecular mechanism driving the effect of NF-κB on MMP-2 needs to be further investigated.

In this study, downregulation of *IκBα* resulted in the decrease of TIMP-2 and the increase of MT1-MMP. TIMP-2 has been found to exhibit several biochemical and physiologic/biological functions including inhibition of active MMPs, pro-MMP activation, and so on. The balance between TIMP-2 and MMP-2 levels is critical in determining the activation status of MMP-2. Along with the results of Figure 4, we found that TIMP-2 expression was reduced while MMP-2 activation was enhanced after *IκBα* siRNA transfection in HCM cells. In addition, activation of MMP-2 also requires its cell surface localization and cleavage by cell membrane-bound MT1-MMP, the increased expression of MT1-MMP promoted the MMP-2 expression. The results showed that increased pro-MMP-2 activation reflected upregulation of MT1-MMP expression in the *IκBα* siRNA treated cells. In a word, the number of function attributed to being regulated by the transcription factor, NF-κBp65, is rapidly increasing. We observed that translocation of NF-κBp65 into the nucleus correlates with the decrease of TIMP-2 and the increase of MT1-MMP. Moreover, the activated NF-κB induced the secretion and activation of MMP-2. This evidence indicated that the expression of MMP-2 is mediated by the activation of NF-κB and that the IκB/NF-κB signaling pathway plays a significant role in this process.

As generally recognized, Rel/NF-κB transcription factors appear to mediate survival signals that protect cells from apoptosis, which may result in the development of several tumors. It has also become clear that MMPs not only play a role in ECM modification but they also have effects on tumor cell adhesion and motility. In our study, the activated NF-κB and consequently, enhanced MMP-2 most likely exert an anti-apoptotic action, promoting the survival of defective cells, thereby causing tumor growth in HCM cells. However, not all reports support a survival role for Rel/NF-κB factors. Experiments by Grimm et al. [33] indicated that a dominant-negative RelA mutant prevented the cell death that was induced upon serum starvation of 293 cells. Jung et al. [34] reported that radiation-induced apoptosis of ataxia telangiectasia (AT) fibroblasts was found to be reduced by a transdominant-negative IκBα protein. Kasibhatla et al. [35] found that inhibition of NF-κB activity in a T cell hybridoma leads to decreased apoptosis in T cells. We have also discovered a few studies reporting the detrimental effects of MMP-2 on HCM cells. Therefore, we intend to shift our experimentation from a predominantly in vitro system to an animal model to further explore the role of the IκB/NF-κB signaling pathway.

In summary, we demonstrated that the IκB/NF-κB signaling pathway plays an important role in the expression of MMP-2 in HCM cells. It is possible that the mechanism underlying the uveoscleral outflow pathway involves the inhibition of IκBα, leading to the activation of NF-κB p65, which in turn, results in the increase of secretion and activity of MMP-2. This molecular mechanism may provide a novel insight in the development of a new strategy for the treatment of glaucoma.
